# Provably Secure Receiver-Unrestricted Group Key Management Scheme for Mobile Ad Hoc Networks †

**DOI:** 10.3390/s23094198

**Published:** 2023-04-22

**Authors:** Rui Zhang, Wendie Han, Lei Zhang, Lulu Wang, Xinyu Meng

**Affiliations:** 1 Shanghai Key Laboratory of Trustworthy Computing, Software Engineering Institute, East China Normal University, Shanghai 200062, China; 52184501020@stu.ecnu.edu.cn (R.Z.); 51194501043@stu.ecnu.edu.cn (W.H.); 52194501009@stu.ecnu.edu.cn (L.W.); 2 Guangxi Key Laboratory of Cryptography and Information Security, Guilin 541004, China; 3 Science and Technology on Communication Security Laboratory, Chengdu 610041, China; 4 Engineering Research Center of Software/Hardware Co-Design Technology and Application, Ministry of Education, East China Normal University, Shanghai 200062, China; 5 College of Information Engineering, Shanghai Maritime University, Shanghai 201306, China; xymeng@shmtu.edu.cn

**Keywords:** mobile ad hoc networks, group key management, security proof, receiver restricted

## Abstract

Mobile ad hoc networks (MANETs) are self-configuring networks of wireless nodes, i.e., mobile devices. Since communications in MANETs occur via wireless channels, it is of significance to secure communications among wireless and mobile nodes. Group key management, as a widely used method for securing group communications, has potentially been used in MANETs for years. Most recently, a secure receiver-unrestricted group key management scheme for MANETs has been proposed, which is used to establish a secure channel among a group of wireless nodes without a trusted dealer, which has some advantages such as eliminating the certificate management problem and receiver restriction. However, a formal security analysis of this scheme is still lacking. Therefore, in this paper, we propose the complete security proof to demonstrate that the scheme satisfies the essential security properties including authentication, message confidentiality, known-key security and dynamic secrecy. We also give a brief discussion about the efficiency of the scheme.

## 1. Introduction

In recent years, mobile ad hoc networks (MANETs) have garnered widespread attention due to their utility and cost-effectiveness. For example, the wireless and mobile nodes in MANETs can still perform effectively in harsh and dynamic environments. This advantage further makes MANETs employed in various fields, including intelligent transportation [1,2], the military field [3], and vehicular ad hoc networks [4]. While enjoying the benefits of MANET, there are still a few security and functional concerns that require our attention. In general, MANETs often consists of numerous mobile and wireless nodes that are responsible for receiving, transmitting and processing data among each other. These interactions among nodes often take place through the wireless communication channel, which makes these mobile and wireless nodes suffer from many attacks, such as impersonation, eavesdropping, forging, and tampering [5]. Hence, it is a challenging task to ensure secure communication in MANETs. Group key management is widely used to establish secure communication channels among wireless nodes by enabling them to exchange encrypted messages through secret keys [6]. Furthermore, the secure channel for wireless communication should support authentication, which ensures that the messages being received are not changed and are coming from a legitimate node. Furthermore, the dynamic feature of MANET makes it difficult to ensure that the established channel remains secure all the time.

Existing group key management schemes for MANETs are realized based on two types of primitives, namely group key distribution (GKD) [7,8,9,10,11] and group key agreement (GKA) [12,13,14,15]. For GKD-based schemes, a trusted dealer is always needed for establishing a secure channel since it is in charge of generating and distributing group key(s) to each sensor node in a group. A trusted dealer is an online trusted party (e.g., a base station), which is often used to authenticate nodes. Our scheme is based on an authenticated scheme, i.e., our identity-based authenticated dynamic contributory broadcast encryption scheme in Section 5. A node is implicitly authenticated in our scheme. Therefore, a trusted dealer (e.g., a base station) is not used to authenticate nodes in our scheme). However, we note the over-dependency of a trusted dealer on increasing the risk of suffering a single-point attack. We also note that, generally, no trusted dealer exists in self-organizing networks such as MANETs. Even if GKA-based schemes eliminate the need for a trusted dealer, receiver restriction still exists, which means that a message from a sender can only be sent to all nodes in the group. This is obviously undesirable in real-world applications (e.g., MANETs) where a sender should be given the rights to choose its preferred sensor nodes within a group to receive the message.

Contributory broadcast encryption (ConBE) [16] can be potentially used to overcome receiver restriction. ConBE can enable wireless nodes to form a group by negotiating a public group encryption key and each wireless node’s decryption key. More importantly, it can allow any node with knowledge of the group encryption key to send encrypted messages to any subset of nodes within the group. Only the selected node can then decrypt the message using its own decryption keys. We note that the first dynamic ConBE scheme was proposed in [17]. However, the scheme is based on traditional public-key cryptography and therefore faces issues with certificate management. Moreover, existing ConBE schemes cannot consider whether the message sent by each wireless node is authenticated. This could potentially increase the risk of an adversary modifying the message.

## 2. Our Contribution

This paper is an extended version of our paper published in 2022 IEEE Wireless Communications and Networking Conference (WCNC) [18], in which an identity-based authenticated dynamic contributory broadcast encryption (IBADConBE) scheme was first discussed. This scheme was further utilized to achieve a secure and receiver-unrestricted group key management scheme for MANETs and could solve all the challenges mentioned in Section 1. In this paper, we first reviewed the original scheme, which has the following advantages. It first allows multiple wireless nodes to dynamically form a group by negotiating a public group encryption key and each node’s decryption key. After that, any sender knowing the public group encryption key can flexibly choose its interesting wireless nodes in the group to receive a message. Additionally, the scheme avoids the issue of certificate management and the need for a trusted dealer. However, in the original work, the authors briefly discussed the security of the scheme without providing a detailed security analysis. Therefore, in this paper, we made an effort to enhance the original work by reviewing it and presenting a formal security proof for it. The additional security analysis and proof contribute to the persuasiveness and trustworthiness of the protocol. We note that there are many schemes (without formal security proof) that were claimed to be secure and later found to be insecure [19]. To this end, we first design the security model for the original IBADConBE scheme. Based on this security model, we give the complete security proof of the IBADConBE scheme, which is based on the asymmetric variant of the decision *k*-Bilinear Diffie-Hellman exponent (BDHE) problem. Formal security proof shows that our scheme satisfies all the desirable security properties, including authentication, message confidentiality, known-key security, and dynamic secrecy, as defined in Section 4.2. In terms of efficiency experiments, in the original work, the authors provided a comprehensive analysis of the computational complexity, communication complexity, and simulations about the running time of each algorithm. According to the simulations, the overall costs are acceptable. Therefore, in this paper, we only briefly summarize the experimental results of the original work.

## 3. Related Work

Secure group communication in MANETs has become an important research topic in recent years, with group key management schemes being the most commonly used method. These schemes can be classified as either group key distribution- (GKD) or group key agreement (GKA)-based, depending on whether they rely on a trusted dealer to generate and distribute the group key(s).

Existing GKD-based schemes for secure communications in MANETs, such as those presented in [7,8,9,10,11], can be categorized into flat and hierarchical types based on network topology. The flat ones include those in [7,8,10,11]. Among them, in [7], an entity called a ground control station (GCS) was employed to distribute a group key and dynamically update the group key when the group membership changes after a fixed period. In [8], a group key pre-distribution scheme was introduced that was used to construct a secure channel among a group of sensors. In [10], the author introduced a symmetric secret key management protocol for multicast communication in MANETs of which a cluster header is selected from a group of sensor nodes and acts as a trusted dealer of the group. Furthermore, a secured key distribution technique was used in the protocol of [11] which was based on key count to effectively distribute the shared pair of keys between two nodes in MANETs. The one in [9] is a hierarchically distributed group key, the management of which is combined with the integrated approach of fuzzy trust-based clustering, which provides an efficient way for group key refreshment in MANETs. In conclusion, the main feature of these schemes is that a trusted dealer is needed to generate and distribute a group key to all nodes in the group. Whenever a node joins or leaves the group, the current group key must be discarded and a new group key should be generated and distributed by the trusted dealer. This results in significant computation and communication overheads, especially making them inefficient for large groups.

To eliminate the need for a trusted dealer, GKA-based group key management schemes were proposed for MANETs, such as the one presented in [12]. Considering the dynamic nature of MANETs, the dynamic scheme was also developed in [13]. However, existing GKA-based schemes still have some limitations, including the requirement for at least two communication rounds to negotiate a secret key, the need for wireless nodes to be online during negotiation, and the inability to allow outside senders to send encrypted messages to a group without first joining the group as a member. Asymmetric group key agreement (AGKA), as a novel group key management technology, was proposed to solve the above problems faced by traditional GKA-based schemes [20]. Later on, AGKA was extended to a new notion called a contributory broadcast encryption (ConBE) [16,21]. Both AGKA and ConBE can enable a group of wireless nodes to negotiate a public group encryption key and each node’s decryption key only in one-round interactions. In contrast to AGKA, ConBE can avoid receiver restriction. Recently, a ConBE scheme for dynamic groups was proposed [17], whose variant designed under the asymmetric group setting was discussed in [22] with lower computation and communication overheads. However, all the ConBE schemes above are designed in the traditional PKI-based cryptosystem. Hence, the burdensome certificate management problem still exists.

## 4. Background

### 4.1. System Architecture

**Figure 1:**
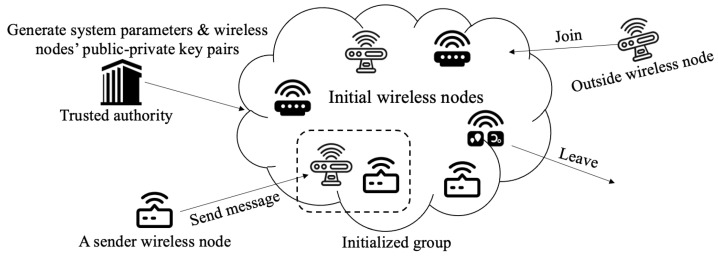
System architecture.

The IBADConBE scheme is mainly for mobile ad hoc networks, in which the nodes are assumed to have a relatively sufficient computation capability. Figure 1 shows the system architecture, which involves a trusted authority (TA), a group of wireless nodes, and a sender (outside the group). As shown in Figure 1, the TA is responsible for generating and publishing the global system parameters. Meanwhile, each wireless node has to enroll with TA and ends up obtaining a public–private key pair issued by TA. We note when a group of wireless nodes that want to form a group, they first need to negotiate a group size (the maximum number of wireless nodes in the group) and then agree on an initial public group encryption key and each node’s individual decryption key. The dynamic nature of MANETs allows outside or inside wireless nodes to join or leave a group at any time. Additionally, a sender who has learned a public group encryption key can send an encrypted messages for some and/or all wireless nodes of a group it favors via a public channel. Furthermore, only those wireless nodes chosen by the sender can read the message.

### 4.2. Design Goals

The design goals that the IBADConBE scheme has achieved can be categorized into security goals and function goals. The security goals include authentication, message confidentiality, known-key security, and dynamic secrecy while the function goals contain trusted dealer freeness, receiver non-restriction, certificate freeness, and dynamicity.

- Authentication: This security goal is used to ensure the legitimacy of wireless nodes in MANETs. That is, all the transmitted messages through communication channels are from legitimate wireless nodes and not altered by an attacker.
- Message confidentiality: This goal is used to ensure that the sent message is only read by those wireless nodes who are chosen by a sender as receivers.
- Known-key security: This goal means that even if an adversary learns some wireless node’s individual decryption keys within a group corresponding to a certain session, they will not obtain the decryption keys held by wireless nodes corresponding to other sessions, especially the target session.
- Dynamic secrecy: This goal is used to secure communication if the group membership of a group has changed. Specifically, when a wireless node permanently leaves a group, it will not know any message subsequently sent to other nodes who still exist in the group; if an outside wireless node joins a group, it cannot learn any message previously delivered to existing inside wireless nodes.

The following ones are function goals that are essential for MANETs.

- Trusted dealer freeness: There is not any trusted dealer needed for generating and distributing secret group key(s) to each wireless node who form a group.
- Receiver non-restriction: Any sender who has known the public group encryption key of a group is allowed to flexibly select its favorable wireless nodes within the group to receive the encrypted message.
- Certificate freeness: There is no need to issue a public key certificate to each wireless node in MANETs to guarantee its legitimacy. Instead, the identity of each node is its public key, which solves the certificate management problem in PKC.
- Dynamicity: Any outside/inside wireless node is allowed to join/leave a group at any moment once the group has been formed.

We note that the TA used in this paper is different from a trusted dealer that is required to be online. The TA can be online or offline in this paper, and is primarily responsible for generating system parameters and issuing a private key to each legitimate node at the enrollment stage. The TA should be online during the GlobeSetup stage and the enrollment stage, since the TA is involved in these two stage. At the other stages, the TA is not involved. Therefore, we say that the TA could be offline.

We also note that the IBADConBE eliminates the usage of a trusted dealer since no online trusted party is employed to generate and distribute the session key to a group of wireless nodes. This can obviously be distinguished from those group key-distributed-based (GDK-based) key management protocols. In GDK-based ones, a trusted dealer often participates in forming a group and may be a base station. We note that, in ad hoc networks, such as mobile ad hoc networks, it is usually assumed that these no trusted dealer-like base station exists. The TA used in this scheme simply generates system parameters and public–private key pairs but does not interfere with the process of group key agreement.

## 5. Review of IBADConBE Scheme

In this section, we first review the IBADConBE scheme proposed in [18] for MANETs.

### 5.1. High-Level Description

The IBADConBE scheme in [18] consists of the four following stages: GlobeSetup, Enrollment, Group Initialization and Maintenance, and Secure Group Communication. At the first stage, TA generates the global system parameters that will be used for the next three stages. At the Enrollment stage, TA issues a public–private key pair for each wireless node in MANETs so that the legitimacy of each wireless node could be guaranteed. In particular, upon the input of the identity of any wireless node (which is also the public key of this node), TA will generate the private key corresponding to the identity of the node using the master secret key. The Group Initialize and Maintenance stage consists of three algorithms in total, respectively, namely Initialize, Join and Leave. The first algorithm is used to initialize a group for a group of wireless nodes with an initial negotiated group encryption key and each wireless node’s decryption key. Both Join and Leave algorithms are used to maintain the secure communication channel whenever the membership of a group has changed. Precisely, once an inside/outside wireless node leaves/joins a group, the public group encryption key and the rest of the nodes’ decryption keys will only update with one-round interaction. Furthermore, we note that during the Group Initialize and Maintenance and Group Communication stages, the TA is an offline trusted party. The Secure Group Communication stage is used to establish a secure channel between any sender and some or all wireless nodes within a group. Specifically, any wireless node (even outside a group) can be a sender since each group’s group encryption key is publicly accessible. More importantly, a sender is able to select its preferred wireless nodes within a group as receivers, and only selected wireless nodes can read the message. Then, we show each stage of the IBADConBE scheme in detail.

### 5.2. GlobeSetup

TA has to generate the global system parameters Δ at this stage as follows: choose three cyclic multiplicative groups $G_{1}$, $G_{2}$, $G_{T}$ with prime order *q*, where $G_{1}$, $G_{2}$ has its generator *g* and $g^{\prime }$, respectively; choose an asymmetric bilinear map $\hat{e}:G_{1}\times G_{2}⟶G_{T}$; choose $\kappa \in Z_{q}^{*}$ as the master secret key and set $g_{pub}=g^{\prime \kappa }$ as its master public key; choose two hash functions $H_{1}$, $H_{2}:{\left\{0,1\right\}}^{*}⟶G_{1}$; choose a secure identity-based signature scheme IDS and a symmetric encryption algorithm $E_{K}\left(\cdot \right)/D_{K}\left(\cdot \right)$. In the IBADConBE scheme, it is assumed that $ID_{\gamma }$ denotes the identity of TA and the corresponding private key is $s_{\gamma }=id_{\gamma }^{\kappa }$, where $id_{\gamma }=H_{1}\left(ID_{\gamma }\right)$. We note that $ID_{\gamma }$ and $s_{\gamma }$ are used to generate *N* tuples consisting of the final Δ. The *N* tuples has the format $\left(f_{\theta },R_{\theta },F_{\theta }\right)$. Each tuple corresponds to a group with the optional group size *n*, which is generated as follows:

1. For 1≤i≤n, choose $r_{i\theta }\in Z_{q}^{*}$ at random and compute $R_{i\theta }=g^{\prime r_{i\theta }}$, $f_{j}=H_{2}\left(j\right)$.
2. For 1≤i,j≤n,i≠j, compute $F_{ij\theta }=s_{\gamma }f_{j}^{r_{i\theta }}$.
3. Set $R_{\theta }={\left\{R_{i\theta }\right\}}_{i\in \left\{1,\ldots ,n\right\}}$, $f_{\theta }={\left\{f_{i}\right\}}_{i\in \left\{1,\ldots ,n\right\}}$ and $F_{\theta }={\left\{F_{j\theta }\right\}}_{\left\{1\leq j\leq n\right\}}$, where $F_{j\theta }={\left\{F_{ij\theta }\right\}}_{\left\{1\leq i\leq n,i\neq j\right\}}$.
4. Obtain and publish the global system parameters $\Delta =(q,g,g^{\prime },G_{1},G_{2},G_{T},g_{pub},H_{1},H_{2},IDS,{\left\{(f_{\theta },R_{\theta },F_{\theta })\right\}}_{\theta \in \left\{1,\ldots ,N\right\}},E_{K}\left(\cdot \right)/D_{K}\left(\cdot \right))$.

### 5.3. Enrollment

In our scheme, each wireless node is required to register with the TA. At this stage, the TA generates private–public key pairs for wireless nodes. It takes master-secret κ and an wireless node’s identity $ID_{i}\in {\left\{0,1\right\}}^{*}$ as input. The public key of a wireless node is set to be its identity. Meanwhile, it computes the private key of the wireless node as follows:

1. Compute $id_{i}=H_{1}\left(ID_{i}\right)$.
2. Compute the private key of the wireless node $s_{i}=id_{i}^{\kappa }$.

Certificates are not a requisite to bind the wireless node’s identities and public keys. Thus, our scheme captures certificate freeness.

### 5.4. Group Initialization and Maintenance

This stage consists of three algorithms (Initialize, Join, Leave), which are used to initialize a group and then dynamically maintain the group. Specifically, a group of wireless nodes who want to form a group first perform the Initialize algorithm to negotiate an initial group encryption key and each wireless node’s decryption key. A suitable group size for the initialized group can be negotiated by all of wireless nodes according to historical experience and the context of applications. After initializing a group, any outside/inside wireless node is allowed to join/leave the group at any time which achieves dynamicity. We note that once that the membership of the group has changed, and the group keys (e.g., group encryption key and each existing wireless node’s decryption) must be updated. In the Join/Leave algorithm, one-round communication is only needed to complete updating.

Initialize: Assume there are *t* wireless nodes ($U_{1},\ldots ,U_{t}$) who want to form a group with the negotiable group size *n*, the corresponding tuple is denoted as $\left(f_{\theta },R_{\theta },F_{\theta }\right)$. For 1≤i≤t, the *i*-th wireless node $U_{i}$ with the public–private key pair $\left(id_{i},s_{i}\right)$ performs as follows:

1. Randomly chooses $r_{i}\in Z_{q}^{*}$ and computes $R_{i}=g^{\prime r_{i}}$.
2. For 1≤j≤n, computes $F_{ij}=s_{i}f_{j}^{r_{i}}$.
3. Sets $M_{i}=\left(ID_{i},R_{i},{\left\{F_{ij}\right\}}_{j\in \left\{1,\ldots ,n\right\},j\neq i}\right)$ and signs $M_{i}$ to obtain a signature $\Upsilon_{i}$ using the ID-based scheme IDS.
4. Publishes $M_{i}=\left(M_{i},\Upsilon_{i}\right)$.

For each wireless node $U_{i}$, it will capture t−1 message–signature pairs ${\left\{M_{k}\right\}}_{1\leq k\leq t,k\neq i}$ from other t−1 wireless nodes, which will be used by $U_{i}$ to calculate the group encryption and its decryption key as follows:

1. Check whether the t−1 message–signature pairs ${\left(M_{i},\Upsilon_{i}\right)}_{1\leq k\leq t,k\neq i}$ are valid. If valid, go to the next step; otherwise, abort.
2. Compute the group encryption key $\left(\hat{E},\hat{\Omega }\right)$, where $\hat{E}=\prod_{l=1}^{t}R_{i}\prod_{l=t+1}^{n}R_{i\theta }$ and $\hat{\Omega }=\hat{e}\left(H_{1}{\left(ID_{\gamma }\right)}^{n-t}\prod_{j=1}^{t}H_{1}\left(ID_{j}\right),g_{pub}\right)$.
3. For 1≤l≤n, obtain ${\hat{S}}_{l}=\prod_{j=1}^{t,j\neq l}F_{jl}\prod_{j=t+1}^{n}F_{jl\theta }$ which are intermediate values to compute the decryption key.
4. Compute the decryption key $S_{i}={\hat{S}}_{i}F_{ii}$, and checks whether Equation (1) holds. If not, it is aborted.
  (1) $$\hat{e}\left(S_{i},g^{\prime }\right)\overset{?}{=}\hat{e}\left(f_{i},\hat{E}\right)\cdot \hat{\Omega }$$
5. Let st be an *n*-bit all zero string. It is used to record the index of free positions in the group. Assume that st[l] denotes the *l*-th bit of st. For 1≤l≤t, set st[l]=1. If ${\left[st\right]}_{l}=1$, it indicates that there exists a wireless node in the position with index *l* of the group.
6. Generate the group member information ${\hat{M}}_{i}=\left\{M_{i};{\hat{S}}_{1},\ldots ,{\hat{S}}_{n};\left(\hat{E},\hat{\Omega }\right);st;S_{i}\right\}$.

Join: Suppose an outside wireless node $U_{I}$ with the public–private key pair $\left(ID_{I},s_{I}\right)$, $s_{I}=H_{1}{\left(ID_{I}\right)}^{\kappa }$ plans to join a group as the *i*-th group member. This requires that st[i]=0, which means that the *i*-th position of the group is free. $U_{I}$ first does the following:

1. Randomly chooses $r_{i}\in Z_{q}^{*}$ and compute $R_{i}=g^{\prime r_{i}}$.
2. For 1≤j≤n, computes $F_{ij}=s_{I}f_{j}^{r_{i}}$.
3. Sets $M_{i}=\left(ID_{I},R_{i},{\left\{F_{ij}\right\}}_{j\in \left\{1,\ldots ,n\right\},j\neq i}\right)$ and generates a signature $\Upsilon_{i}$ by using IDS.
4. Publishes $M_{i}=\left(M_{i},\Upsilon_{i}\right)$.

In the sequel, each existing wireless node in the group will receive the message $M_{i}=\left(M_{i},\Upsilon_{i}\right)$ from $U_{i}$. For any wireless node in the group (assume that *j*-th satisfies $j\in \left\{k|st[k]=1\right\}$), it does the following:

1. Check whether the message–signature pair $\left(M_{i},\Upsilon_{i}\right)$ is valid. If not, it is aborted; otherwise, the next step ensues.
2. For 1≤l≤n,l≠i, update ${\hat{S}}_{l}={\hat{S}}_{l}F_{il}F_{il\theta }^{-1}$.
3. Update $\hat{E}=\hat{E}R_{i}R_{i\theta }^{-1}$, $\hat{\Omega }=\hat{\Omega }\cdot \hat{e}\left(H_{1}\left(ID_{I}\right),g_{pub}\right)\cdot \hat{e}{\left(H_{1}\left(ID_{\gamma }\right),g_{pub}\right)}^{-1}$ and $S_{j}={\hat{S}}_{j}F_{ij}F_{ij\theta }^{-1}$.
4. Check whether Equation (1) holds. If Equation (1) does not hold, it is aborted.
5. Set st[i]=1 and the new member information ${\hat{M}}_{i}=\left\{M_{i};{\hat{S}}_{1},\ldots ,{\hat{S}}_{n};\left(\hat{E},\hat{\Omega }\right);st;S_{i}\right\}$.

We note that, for the new group member $U_{I}$, it requires messages $\left\{{\hat{S}}_{1},\ldots ,{\hat{S}}_{n};\left(\hat{E},\hat{\Omega }\right);st\right\}$ to compute its decryption key $S_{i}$. Hence, it is assumed that the wireless node with the minimal index of the group has to deliver $\left\{{\hat{S}}_{1},\ldots ,{\hat{S}}_{n};\left(\hat{E},\hat{\Omega }\right);st\right\}$ to $U_{I}$. After receiving the above message, $U_{I}$ does the following to obtain its member information:

1. Computes the decryption key $S_{i}={\hat{S}}_{i}F_{ii}$ and check whether Equation (1) holds. If not, it is aborted; otherwise, the next step ensues.
2. Stores the group member information ${\hat{M}}_{i}=\left\{M_{i};{\hat{S}}_{1},\ldots ,{\hat{S}}_{n};\left(\hat{E},\hat{\Omega }\right);st;S_{i}\right\}$.

Leave: Assume an inside wireless node $U_{I}$ as the *i*-th group member wants to leave the group. It does as follows:

1. Lets $M_{i}^{\prime }=M_{i}$ and generate a new signature $\Upsilon_{i}^{\prime }$ on $M_{i}^{\prime }$ using IDS.
2. Publishes $M_{i}^{\prime }=\left(M_{i}^{\prime },\Upsilon_{i}^{\prime }\right)$.

After obtaining the message–signature pair $M_{i}^{\prime }=\left(M_{i}^{\prime },\Upsilon_{i}^{\prime }\right)$ from $U_{I}$, each existing wireless node in the group will use it to update their member information. For the *j*-th group member ($j\in \left\{k|st[k]=1,k\neq i\right\}$), it does the following:

1. Checks whether the message–signature pair $\left(M_{i}^{\prime },\Upsilon_{i}^{\prime }\right)$ is valid. If not, it is aborted; otherwise, the next step ensues.
2. For 1≤l≤n,l≠i, updates ${\hat{S}}_{l}={\hat{S}}_{l}F_{il}^{-1}F_{il\theta }$.
3. Updates $\hat{E}=\hat{E}R_{i\theta }R_{i}^{-1}$, $\hat{\Omega }=\hat{\Omega }\cdot \hat{e}\left(H_{1}\left(ID_{\gamma }\right),g_{pub}\right)\cdot {\left(H_{1}\left(ID_{I}\right),g_{pub}\right)}^{-1}$ and $S_{j}={\hat{S}}_{j}F_{ij}^{-1}F_{ij\theta }$.
4. Checks whether Equation (1) holds. If Equation (1) does not satisfy, it is aborted.
5. Sets st[i]=0 and updates and stores new member information ${\hat{M}}_{i}$.

We note that, during the whole stage of Group Initialization and Maintenance, the group encryption key and each wireless node’s decryption key are generated through the negotiation of wireless nodes themselves, instead of relying on a trusted dealer to generate and distribute these group keys. Hence, this scheme achieves the design goals of trusted dealer freeness.

### 5.5. Secure Group Communication

At this stage, a sender can securely transmit a message to any wireless nodes that the sender selects from a group based on its preference. There are two algorithms included at this stage, respectively, Encrypt and Decrypt. Any sender who has the knowledge of the group encryption key of a group first selects some wireless nodes that it wants to communicate with and then generates a ciphertext by running the Encrypt algorithm. The wireless nodes within the group which are selected by the sender as receivers are able to decrypt the ciphertext and read the message by performing the Decrypt algorithm.

Encrypt: Assume that a sender wants to send the message *m* to some wireless nodes in a group and the selected wireless nodes within the group form an index set denoted by U. Let $S=\left\{k|st[k]=1\right\}$, $\bar{S}=\left\{{i|\left[st\right]}_{i}=0\right\}$, $\bar{U}=S\setminus U$. To obtain the final ciphertext, the sender performs the following steps:

1. Computes $\Omega =\hat{\Omega }\cdot \prod_{l\in \bar{U}}^{}\hat{e}\left(H_{1}\left(ID_{\gamma }\right),g_{pub}\right)$, $E=\hat{E}\cdot \prod_{l\in \bar{U}}^{}R_{l\theta }$.
2. Randomly chooses $a\in Z_{q}^{*}$, computes the ciphertext $C=(C_{1},C_{2},C_{3})$, where $C_{1}=g^{\prime a}$, $C_{2}=E^{a}$, $C_{3}=E_{sk}\left(m\right)$, and the session key is
  $$sk=\Omega^{a}=\hat{e}{\left(\prod_{k\in S}^{}s_{k}\prod_{k\in \bar{S}}s_{\gamma }\prod_{k\in \bar{U}}s_{\gamma },g^{\prime }\right)}^{a}$$
3. Sends (C,U) to the group.

Decrypt: Only wireless nodes in U are capable of decrypting the above ciphertext and then extract the session key sk and read the message *m*, which captures the receiver non-restriction. For each wireless node in U, (assume that the *i*-th wireless node i∈U), it does the following:

1. Computes ${\tilde{S}}_{i}=S_{i}\prod_{l\in \bar{U}}^{}F_{li\theta }$ and then computes the session key
  $$\begin{array}{cc} sk= & \hat{e}\left({\tilde{S}}_{i},C_{1}\right)\hat{e}{\left(f_{i},C_{2}\right)}^{-1}\\ = & \hat{e}{\left(\prod_{k\in S}^{}s_{k}\prod_{k\in \bar{S}}s_{\gamma }\prod_{k\in \bar{U}}s_{\gamma },g^{\prime }\right)}^{a} \end{array}$$
2. Computes $m=D_{sk}\left(C_{3}\right)$.

## 6. Security

### 6.1. Security Model and Definitions

The IBADConBE scheme captures authentication, message confidentiality, known-key security, and dynamic secrecy, of which authentication was ensured by a secure identity-based signature scheme. Thus, we only have to prove that the IBADConBE scheme captures the remaining security properties. Firstly, we give the security model for the IBADConBE scheme, which is the security game run between a challenger C and an adversary A. In this game, C plays the role of TA, generates the system-wide parameters and answers different types of queries from A. Our security model consists of four stages: **Initialize**, **Attack**, **Challenge**, and **Response**. The first and second stages simulate each algorithm of IBADConBE. Meanwhile, at the **Attack** stage, an adversary is allowed to make various queries, which simulates various attack behaviors. For instance, Corrupt and CorruptKey queries model the leakage of private keys and random coins held by users, Reveal queries model the disclose of session keys (corresponding to the known-key attack), and Join/Leave models the attacker controlling a node to join/leave a group. At the **Challenge** stage, the adversary submits ($m_{0},m_{1}$) and obtains a challenge ciphertext *c* (generated from $m_{0}$ or $m_{1}$). However, at the last stage, the advantage of the adversary to guess that *c* is from $m_{0}$ or $m_{1}$ is still negligible, even when the advantage can make Reveal, Join and Leave queries. Therefore, the IBADConBE proves to capture message confidentiality, known-key security, and dynamic secrecy.

**Initialize**: C generates the system-wide parameters Δ by running the GlobeSetup algorithm and passes it to A.

**Attack**: C answers the following queries from A:

- Execute(t,n): This query is used to model the initialize algorithm at the group initialization and maintenance stage. A submits (t,n), where *t* and *n* denote the number of initial participants and group size A selects. C initializes a group, with a unique index μ, and sets the initial session ID η to be 1. η should be set to η+1 if A invokes the following Join(i,μ) or Leave(i,μ) query.
- Join(i,μ): This query is used to model the joint algorithm at the group initialization and maintenance stage. Upon receiving this query, C enables an outside node to join the group with the index μ as the *i*-th group member. This query can be asked for at most *K* times.
- Leave(i,μ): This query is used to model the Leave algorithm at the group initialization and maintenance stage. Upon receiving this query, C enables the *i*-th inside node in the μ-group to leave permanently.
- CorruptKey$\left(ID_{i}\right)$: Upon receiving this query, C outputs the private key held by $ID_{i}$. This query can be used to model (partial) forward secrecy.
- Corrupt(i,μ,η): Upon receiving this query, C outputs the private input and/or inner random coins held by the *i*-th inside node corresponding to the η-th session of the μ-th group.
- Reveal(i,μ,η): Upon receiving this query, C outputs the decryption key held by the *i*-th inside node corresponding to the η session in the μ-th group. This query can be used to model known-key security.

**Challenge**: At this stage, A submits $\left\{U^{*},\mu^{*},\eta^{*},\left(m_{0},m_{1}\right)\right\}$ to C, where $U^{*}\subseteq K=\left\{1,\ldots ,K\right\}$ is a fresh set (see Definition 1), $\mu^{*}$, $\eta^{*}$ is the index of the target group and the target session ID, $\left(m_{0},m_{1}\right)$ is a pair of messages with the same length. C randomly chooses a bit $b\in \left\{0,1\right\}$. If b=0, C returns the challenge ciphertext $C^{*}$ generated from encrypting $m_{0}$; otherwise, C returns the ciphertext $C^{*}$ by encrypting $m_{1}$.

**Response**: At this stage, A returns a guess $b^{\prime }\in \left\{0,1\right\}$. If $b^{\prime }=b$, A wins the game. A’s advantage to win the above game is defined as $\mathrm{Adv}\left(A\right)=\left|\mathrm{Pr}[b=b^{\prime }]-1\right|$.

**Definition** **1** (Freshness). A set $U^{*}$ is fresh if none of the following conditions are satisfied: (1) A has made a Reveal(i,μ,η) query on any node with index in $U^{*}$ within the target group; (2) A has made Corrupt(i,μ,η) queries on any node with the index in $U^{*}$; (3) All the private keys of the nodes participating in the target session of target group are corrupted.

**Definition** **2.** An IBADConBE scheme is said to be fully and adaptively secure against chosen plaintext attacks (CPA) if no polynomial-time adversary A can win the above game with an advantage Adv(A). An IBDConBE scheme is said to be semi-adaptively secure if the adversary (1) has to commit an index set K before the Attack stage; (2) can only choose $U^{*}\subseteq K$ to query C at the challenge stage.

We note that A cannot successfully distinguish $C^{*}$ comes from $m_{0}$ or $m_{1}$, even when A is allowed to ask CorruptKey$\left(ID_{i}\right)$ and Corrupt(i,μ,η) queries for any node (not in $U^{*}$). This further implies that A cannot violate the confidentiality of an encrypted message in the real world. Thus, the scheme captures the message confidentiality. Additionally, A is allowed to reveal some nodes’ decryption keys that do not correspond to the target session of the target group. Thus, the scheme satisfies the capture of the known-key security. At the end of **Challenge** stage, A is allowed to invoke Join/Leave queries, but its advantage to win the game is still negligible. Hence, the scheme satisfies dynamic secrecy.

### 6.2. Security Proof

**Theorem** **1.** *Let $H_{1}$, $H_{2}$ be random oracles. Suppose that C may initialize at most N groups and L sessions for each group, the maximal group size is k, and A made at most $q_{H_{1}}$ queries to $H_{1}$ oracle. If the A wins the above game with the advantage Adv(A) in time τ, there exists an algorithm to solve the asymmetric variant of the decision k-BDHE problem with an advantage at least $\frac{1}{NLq_{H_{1}}}Adv\left(A\right)$ in time $\tau +O\left(kNL\right)\tau_{E}$, where $\tau_{E}$ computes a scalar multiplication in $G_{1}$.*

Asymmetric variant of a decision *k*-BDHE problem: Given a bilinear map: $\hat{e}:G_{1}\times G_{2}\to G_{T}$, $P=g^{p}$, $Q=g^{\prime h}$, $X={\left\{x_{i}=g^{\alpha^{i}}\right\}}_{\left\{i=1,2,...,k,k+2,...,2k\right\}}$, $Y_{1}={\left\{y_{j}=g^{\prime \alpha^{j}}\right\}}_{\left\{j=1,2,...,k+1\right\}}$, for unknown $\alpha ,p,h\in Z_{q}^{*}$. An algorithm D that outputs b∈{0,1} has the advantage ϵ in solving the asymmetric variant of the decision *k*-BDHE problem if

$$\begin{array}{c} |\mathrm{Pr}[D\left(g,g^{\prime },P,Q,X,Y_{1},Z_{0}\right)=0]-\\ \mathrm{Pr}[D\left(g,g^{\prime },P,Q,X,Y_{1},Z_{1}\right)=0]|\geq \epsilon \end{array}$$

where $Z_{0}=\hat{e}\left(g^{\alpha^{k+1}},Q\right)$ and $Z_{1}\in G_{T}$ randomly. The asymmetric variant of the decision *k*-BDHE assumption holds in $G_{T}$ if no polynomial-time algorithm has the advantage of at least ϵ in solving the asymmetric variant of the decision *k*-BDHE problem in $G_{T}$.

**Proof of Theorem** **1.** Let C be a challenge and A be an adversary. C is given an asymmetric variant of the *k*-BDHE problem instance $\left(g,g^{\prime },P,Q,Z,x_{1},...,x_{k},x_{k+2},...,x_{2k},y_{1},...,y_{k}\right)$, where $P\in G_{1},Q\in G_{2}$, $x_{i}=g^{\alpha^{i}},i\in \left\{1,...,k,k+2,...,2k\right\}$, $y_{i}=g^{\prime \alpha^{j}},j\in \left\{1,...,k+1\right\}$ with some unknown $\alpha \in Z_{q}^{*}$. We show how C can utilize A to determine whether *Z* is equal to $\hat{e}\left(g^{\alpha^{k+1}},Q\right)$ or a uniform element in $G_{T}$.

**Initialize**: Assume that two random oracles $H_{1}$ and $H_{2}$ answer queries as follows:

$H_{1}$ queries: C keeps an initially empty list $H_{1}^{list}$. Upon input $ID_{i}$, C performs the following:

- If there is a tuple $\left(ID_{i},\mu_{i},id_{i},s_{i}\right)$, returns $id_{i}$.
- Otherwise, chooses $\mu_{i}\in Z_{q}^{*}$ at random, and if this query is the *J*-th target query, sets $id_{i}=g^{\mu_{i}}$, $s_{i}=x_{k}^{\mu_{i}}$; otherwise, sets $id_{i}=g^{\mu_{i}}$, $s_{i}=x_{1}^{\mu_{i}}$.
- Adds $\left(ID_{i},\mu_{i},id_{i},s_{i}\right)$ to $H_{1}^{list}$ and returns $id_{i}$.

$H_{2}$ queries: C keeps an initially empty list $H_{2}^{list}$. Upon input *j*, C performs the following:

- If there is a tuple $\left(j,v_{j},f_{j}\right)$, returns $f_{j}$.
- Otherwise, randomly chooses $v_{j}\in Z_{q}^{*}$, sets $f_{j}=g^{v_{j}}$, adds $\left(j,v_{j},f_{j}\right)$ to $H_{2}^{list}$ and returns $f_{j}$.

C sets the system-wide parameters $\Delta =(q,g,G_{1},G_{2},g_{pub},H_{1},H_{2},IDS,{\left\{(f_{\theta },R_{\theta },F_{\theta })\right\}}_{\theta \in \left\{1,\ldots ,N\right\}})$, where $g_{pub}=g^{\prime \alpha }=y_{1}$. Assume that $ID_{\gamma }$ denotes the identity of TA. To generate the tuple, $\left(f_{\theta },R_{\theta },F_{\theta }\right)$ corresponding to group size *n*, C first recovers $\left(ID_{\gamma },\mu_{\gamma },id_{\gamma },s_{\gamma }\right)$ from $H_{1}^{list}$ and $\left(j,v_{j},f_{j}\right)$, 1≤j≤n from $H_{2}^{list}$, and then, for 1≤i≤n, performs the following:

- If i=1, performs as follows:
  1. Chooses $r_{1\theta }\in Z_{q}^{*}$ at random and computes $R_{1\theta }=g^{\prime r_{1\theta }}\prod_{l=2}^{n}y_{k-l+1}^{-1}$.
  2. Computes $F_{1j\theta }=x_{1}^{\mu_{\gamma }}g^{v_{j}r_{1\theta }}\prod_{l=2}^{n,l\neq j}x_{k-l+1+j}^{-1}$, 2≤j≤n,
  3. Sets $F_{11\theta }=null$.
- Otherwise, for 2≤i≤n, C performs the following:
  1. Chooses $r_{i\theta }\in Z_{q}^{*}$ randomly and computes $R_{i\theta }=g^{\prime r_{i\theta }}y_{k-i+1}$.
  2. For 2≤j≤n, computes $F_{ij\theta }=x_{1}^{\mu_{\gamma }}g^{v_{j}r_{i\theta }}x_{k-i+1+j}$.
  3. Sets $F_{ii\theta }=null$.

Δ is passed to A. A then commits a set $U\subseteq \left\{1,\ldots ,K\right\}$ to C. Finally, C randomly chooses $\mu \in \left\{1,\ldots ,N\right\}$ and $\omega \in \left\{1,\ldots ,L\right\}$. In the following, we assume that C will answers the queries as in the real scheme if it is not the μ-th group. Hence, we only need to consider the queries from A corresponding to target group.

**Attack**: C answers A’s queries as follows:

Execute(t,n): C maintains an initially empty list $T_{list}$ and sets the initial session ID η=1. Suppose the set of *t* initial participants’ identities is $\left\{ID_{1},\ldots ,ID_{t}\right\}$. To answer this query, C first submits $\left\{ID_{1},\ldots ,ID_{t}\right\}$ to $H_{1}$ if these queries have never been issued before, and then recovers $\left(ID_{i},\mu_{i},id_{i},s_{i}\right)$ for 1≤i≤t from $H_{1}^{list}$. For 1≤i≤t, C generates a coin $coin_{i\eta }$ and then performs the following:

- If i∉K, sets $coin_{i\eta }=0$ and then performs the following:
  1. Chooses $r_{i\eta }\in Z_{q}^{*}$ and sets $R_{i\eta }=g^{\prime r_{i\eta }}$
  2. For 1≤j≤n, computes $F_{ij\eta }=s_{i}f_{j}^{r_{i\eta }}$.
- Otherwise, C sets $coin_{i\eta }=1$. If and only if i=1, then C performs the following:
  1. Chooses $r_{i\eta }\in Z_{q}^{*}$ and computes $R_{i\eta }=g^{\prime r_{i\eta }}\prod_{l=2}^{n}y_{k-l+1}^{-1}$.
  2. For 1≤j≤n, j≠i, sets $F_{ij\eta }=x_{1}^{\mu_{i}}g^{v_{j}r_{i\eta }}\prod_{l=1}^{n,l\neq j}x_{k-l+1+j}^{-1}$.
  3. Sets $F_{ii\eta }=null$.
- Otherwise, C performs the following:
  1. Chooses $r_{i\eta }\in Z_{q}^{*}$ and computes $R_{i\eta }=g^{\prime r_{i\eta }}y_{k-i+1}$.
  2. For 1≤j≤n, j≠i, sets $F_{ij\eta }=x_{1}^{\mu_{i}}g^{v_{j}r_{i\eta }}g_{k-i+1+j}$.
  3. Sets $F_{ii\eta }=null$ and $M_{i\eta }=\left(ID_{i},R_{i\eta },{\left\{F_{ij\eta }\right\}}_{j\in \{1,...,n\},j\neq i}\right)$
  4. Signs $M_{i\eta }$ to obtain a signature $\Upsilon_{i\eta }$ using IDS and publishes $\left(M_{i\eta },\Upsilon_{i\eta }\right)$.

Let ${\bar{M}}_{i\eta }=(coin_{i\eta },ID_{i},r_{i\eta },R_{i\eta },\left\{F_{ij\eta }\right\}$ ${}_{j\in \left\{1,\ldots ,n\right\}})$, and then C performs the following:

1. Computes the public group encryption key $\left({\hat{E}}_{\eta },{\hat{\Omega }}_{\eta }\right)$, where ${\hat{E}}_{\eta }=\prod_{l=1}^{t}R_{i\eta }\prod_{l=t+1}^{n}R_{i\theta }$ and ${\hat{\Omega }}_{\eta }=\hat{e}\left(H_{1}{\left(ID_{\gamma }\right)}^{n-t}\prod_{j=1}^{t}H_{1}\left(ID_{j}\right),g_{pub}\right)$.
2. Computes ${\hat{S}}_{i\eta }=\prod_{j=1}^{t,j\neq i}F_{ji\eta }\prod_{j=t+1}^{n}F_{ji\theta }$ for 1≤i≤n.
3. Lets $st_{\eta }$ be a *n*-bit all-zero string. For 1≤i≤t, sets $st_{\eta }\left[i\right]=1$.
4. Adds $T_{\eta }=\left(\eta ;{\bar{M}}_{1\eta },\ldots ,{\bar{M}}_{t\eta };{\hat{S}}_{1\eta },\ldots ,{\hat{S}}_{n\eta };st_{\eta };{\hat{E}}_{\eta },{\hat{\Omega }}_{\eta }\right)$ to list $T_{list}$.
5. Returns ${\left\{ID_{i},R_{i\eta },\Upsilon_{i},{\left\{F_{ij\eta }\right\}}_{j\in \left\{1,\ldots ,n\right\}\setminus i}\right\}}_{i\in \left\{1,\ldots ,t\right\}}$.

We note that the Execute query can be simulated by invoking the following Join query for *t* times. If C answers the above Execute query, then η is set to *t*.

In the following, if we set $T_{\eta }=T_{\eta -1}$, then C performs the following:

1. Sets $st_{\eta }=st_{\eta -1}$,${\hat{E}}_{\eta }={\hat{E}}_{\eta -1}$,${\hat{\Omega }}_{\eta }={\hat{\Omega }}_{\eta -1}$.
2. Sets ${\bar{M}}_{i\eta }={\bar{M}}_{i(\eta -1)}$ for 1≤i≤t.
3. Sets ${\hat{S}}_{i\eta }={\hat{S}}_{i(\eta -1)}$ for 1≤i≤n.
4. Adds $T_{\eta }=\left(\eta ;{\bar{M}}_{1\eta },\ldots ,{\bar{M}}_{t\eta };{\hat{S}}_{1\eta },\ldots ,{\hat{S}}_{n\eta };st_{\eta };{\hat{E}}_{\eta },,{\hat{\Omega }}_{\eta }\right)$ to list $T_{list}$.

Join(i,μ,η): Assume a node with $ID_{i}$ wants to join the group as the *i*-th group member. C sets $T_{\eta }=T_{\eta -1}$, and then performs the following:

- If i∉K, C sets $coin_{i\eta }=0$ and then performs the following:
  1. Chooses $r_{i\eta }\in Z_{q}^{*}$, set $R_{i\eta }=g^{\prime r_{i\eta }}$
  2. For 1≤j≤n, computes $F_{ij\eta }=s_{i}f_{j}^{r_{i\eta }}$.
- Otherwise, sets $coin_{i\eta }=1$, if i=1, and then performs the following:
  1. Chooses $r_{i\eta }\in Z_{q}^{*}$ and computes $R_{i\eta }=g^{\prime r_{i\eta }}\prod_{l=2}^{n}y_{k-l+1}^{-1}$.
  2. For 1≤j≤n, j≠i, computes $F_{ij\eta }=g_{pub}^{\mu_{i}}g^{v_{j}r_{i\eta }}\prod_{l=1}^{n,l\neq j}x_{k-l+1+j}^{-1}$.
  3. Sets $F_{ii\eta }=null$.
- Otherwise, C performs the following:
  1. Chooses $r_{i\eta }\in Z_{q}^{*}$ and computes $R_{i\eta }=g^{\prime r_{i\eta }}y_{k-i+1}$.
  2. For 1≤j≤n, j≠i, computes $F_{ij\eta }=x_{1}^{\mu_{i}}g^{v_{j}r_{i\eta }}x_{k-i+1+j}$.
  3. Sets $F_{ii\eta }=null$ and $M_{i\eta }=\left(ID_{i},R_{i\eta },{\left\{F_{ij\eta }\right\}}_{j\in \{1,...,n\},j\neq i}\right)$
  4. Signs $M_{i\eta }$ to obtain a signature $\Upsilon_{i\eta }$ using IDS.

C obtains ${\hat{E}}_{\eta }={\hat{E}}_{\eta }R_{i\eta }R_{i\theta }^{-1}$, ${\hat{\Omega }}_{\eta }={\hat{\Omega }}_{\eta }\cdot \hat{e}\left(H_{1}\left(ID_{i}\right),g_{pub}\right)\cdot$, sets ${\hat{S}}_{l\eta }={\hat{S}}_{l\eta }F_{il\eta }F_{il\theta }^{-1}$, 1≤l≤n, l≠i, $st_{\eta }\left[i\right]=1$, adds ${\bar{M}}_{i\eta }=\left(coin_{i\eta },ID_{I},r_{i\eta },R_{i\eta },{\left\{F_{ij\eta }\right\}}_{j\in \left\{1,\ldots ,n\right\}}\right)$ to $T_{\eta }$ and returns $\left(M_{i\eta },\Upsilon_{i\eta }\right)$ and $\left({\hat{S}}_{1\eta },\ldots ,{\hat{S}}_{n\eta };st_{\eta };{\hat{E}}_{\eta },\Omega_{\eta }\right)$.

Leave (i,μ,η): Assume that the *i*-th group member with $ID_{i}$ intends to leave the μ-th group. C performs the following:

1. Sets $T_{\eta }=T_{\eta -1}$.
2. Returns the tuple $\left(ID_{i},R_{i\eta },\Upsilon_{i},{\left\{F_{ij\eta }\right\}}_{j\in \left\{1,\ldots ,n\right\}\setminus i}\right)$ and removes the tuple ${\bar{M}}_{i\eta }$ from $T_{\eta }$.
3. Sets $st_{\eta }\left[i\right]=0$.
4. Sets ${\hat{E}}_{\eta }={\hat{E}}_{\eta }R_{i\eta }^{-1}R_{i\theta }$, ${\hat{\Omega }}_{\eta }={\hat{\Omega }}_{\eta }\hat{e}{\left(H_{1}\left(ID_{i}\right),g_{pub}\right)}^{-1}\cdot$, $\hat{e}\left(H_{1}\left(ID_{\gamma }\right),g_{pub}\right)$.
5. Sets ${\hat{S}}_{l\eta }={\hat{S}}_{l\eta }F_{il\eta }^{-1}F_{il\theta }$ for 1≤l≤n, l≠i.
6. Returns $\left(M_{i\eta },\Upsilon_{i\eta }\right)$, where $M_{i\eta }=\left(ID_{i},R_{i\eta },{\left\{F_{ij\eta }\right\}}_{j\in \{1,...,n\},j\neq i}\right)$, $\Upsilon_{i\eta }$ is the signature on $M_{i\eta }$.

CorruptKey $\left(ID_{i}\right)$: C first submits $ID_{i}$ to the $H_{1}$ oracle if this query has never been asked before, recovers $\left(ID_{i},\mu_{i},id_{i},s_{i}\right)$ from $H_{1}^{list}$ and returns $s_{i}$.

Corrupt (i,μ,η): This query requires $i\in \bar{K}$. C returns $r_{i}$ held by an *i*-th member corresponding to the η-th session of μ-th group.

Reveal (i,μ,η): The query requires $i\in \bar{K}$. C recovers $T_{\eta }$ from $T_{list}$ and ${\bar{M}}_{i\eta }$ from $T_{\eta }$. C recovers ${\hat{S}}_{i\eta }$ from ${\bar{M}}_{i\eta }$ and returns ${\hat{S}}_{i\eta }F_{ii\eta }$.

Challenge: A chooses a target set $U^{*}\subseteq K$ and a target session $\eta^{*}$ corresponding to the target group. Let $T_{\eta^{*}}$ be $\left(\eta^{*};{\bar{M}}_{1\eta^{*}},\ldots ,{\bar{M}}_{t\eta^{*}};{\hat{S}}_{1\eta^{*}},\ldots ,{\hat{S}}_{n\eta^{*}};st_{\eta^{*}};{\hat{E}}_{\eta^{*}};{\hat{\Omega }}_{\eta^{*}}\right)$. We have $S^{*}=\left\{i|st_{\eta^{*}}\left[i\right]=1\right\}$, ${\bar{S}}^{*}=\left\{i|st_{\eta^{*}}\left[i\right]=0\right\}$, ${\bar{U}}^{*}=S^{*}\setminus U^{*}$. We say that Event 1 happens if the group A that submits is not the μ-group or $\eta^{*}\neq \omega$. Furthermore, Event 2 happens if a node exits with index $l^{*}$ that corresponds to the *J*-th $H_{1}$ query. If Event 1 does not happen and Event 2 happens, C performs as follows, otherwise, C aborts.

1. Set $C_{1}^{*}=Q$, $C_{2}^{*}=Q^{\sum_{i\in S^{*}}^{}r_{i\eta^{*}}+\sum_{i\in {\bar{S}}^{*}\cup {\bar{U}}^{*}}^{}r_{i\theta }}$.
2. Set $sk^{*}=\hat{e}{\left(x_{1},Q\right)}^{\sum_{i\in S^{*},i\neq l^{*}}^{}\mu_{i}+\sum_{i\in {\bar{S}}^{*}\cup {\bar{U}}^{*}}^{}\mu_{\gamma }}$.
3. Choose b∈{0,1} and obtain $C_{3}^{*}=E_{sk^{*}}\left(m_{b}\right)$.
4. Return $\left(C_{1}^{*},C_{2}^{*},C_{3}^{*}\right)$.

**Response**: Finally, C returns their guess $b^{\prime }\in \left\{0,1\right\}$.

If C does not abort, the above simulations of all queries are valid and the answers are uniformly distributed. Hence, the adversary cannot find any inconsistency between the simulation and the real world. Therefore, $\mathrm{Pr}[b=b^{\prime }]\geq Adv\left(A\right)$. For our setting, it is easy to have $\mathrm{Pr}[\neg \mathrm{Event}\;1]\geq \frac{1}{NL}$. Furthermore, $\mathrm{Pr}[\mathrm{Event}\;2]\geq \frac{1}{q_{H_{1}}}$, hence, the overall probability for C to solve the asymmetric variant of decision *k*-BDHE problem is at least $\frac{1}{NLq_{H_{1}}}Adv\left(A\right)$. The time complexity is $\tau +O\left(kNL\right)\tau_{E}$. □

## 7. Performance Analysis

### 7.1. Comparison

According to the evaluation work in [18], we also compare the DAGKA scheme in [23] and the DConBE protocol in [17] with the IBADConBE scheme in this paper in terms of design goals and computation overheads. Table 1 and Table 2, respectively, list the comparison results.

We make a little modification regarding the design goals that the IBADConBE scheme achieves (see in Section 4.2). In particular, we replace the forward secrecy and backward secrecy with dynamic secrecy. Furthermore, we use trusted dealer freeness to replace no trusted dealer in [18]. As shown in Table 1, one can see that only the IBADConBE scheme realizes design goals, i.e., authentication, message confidentiality, known-key security, dynamic secrecy, trusted dealer freeness, receiver non-restriction, certificate freeness, and dynamicity.

**Table 2:** Comparison of the computation overheads.

Algorithms	DAGKA [23]	DConBE [17]	IBADConBE
Initialize	$O\left(n\right)t_{e}$+$O\left(n\right)t_{h}$+$O\left(n\right)t_{m}$	$O\left(n\right)t_{e}$+$O\left(n\right)t_{m}$	$O\left(n\right)t_{e}$+$O\left(n\right)t_{m}$
Join	$O\left(n\right)t_{e}$+$O\left(n\right)t_{h}$+$O\left(n\right)t_{m}$	$O\left(n\right)t_{e}$+$O\left(n\right)t_{m}$	$O\left(n\right)t_{e}$+$O\left(n\right)t_{m}$
Leave	$O\left(n\right)t_{e}$+$O\left(n\right)t_{h}$+$O\left(n\right)t_{m}$	$O\left(n\right)t_{m}$	$O\left(n\right)t_{m}$
Encrypt	$2t_{e}$	$3t_{e}$+$t_{E}$	$3t_{e}$+$t_{E}$+$t_{sg}$
Decrypt	$2t_{b}$	$2t_{b}$+$t_{m}$+$t_{e}$+$t_{D}$	$2t_{b}$+$t_{m}$+$t_{e}$+$t_{D}$+$t_{sv}$

In Table 2, one can see the comparison between the IBADConBE scheme with those in [17,23] in terms of the computational overheads. Let $t_{m}$/$t_{e}$ represent the time to compute a scalar multiplication/exponentiation operation in $G_{1}$ or $G_{2}$, $t_{h}$ represent the time to compute a MapToPoint hash [23], and $t_{b}$ represent the time to compute a scalar bilinear map operation. Furthermore, let $t_{E}$/$t_{D}$ denote the time to compute an encryption/decryption operation using a symmetric cryptographic algorithm. $t_{sg}$/$t_{sv}$ denotes the time to generate/verify an identity-based signature. We note that the time of a scalar multiplication operation is trivial in comparison with that of other operations, and some operations that can be pre-computed were ignored. Obviously, the IBADConBE scheme is more efficient than the DAGKA in [23] and has comparable computational overheads with the DConBE in [17].

### 7.2. Simulations

**Figure 2:**
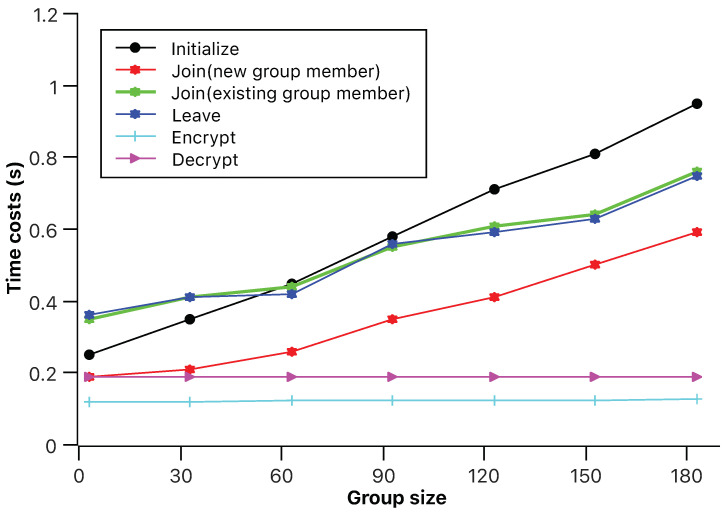
Average time costs.

The main contribution of this paper is to formally prove the security of the IBADConBE scheme in [18]. As for the detailed simulations, they can be found in an experimental part of the work published on 2022 WCNC [18]. Therefore, we only described the simulation results regarding the efficiency of the IBADConBE scheme based on the experimental part of the work published on 2022 WCNC. The settings of the simulations are consistent with those in [18]. In particular, the MIRACL library [22] was used to implement each algorithms of the IBADConBE scheme. The BN curve with a 128-bit security level was selected. The simulations were run on a RaspberryPi 3b+ with an ARM Cortex-A53 CPU at a frequency of 1.4 GHz. The group size was set from 3 to 180. Since GlobeSetup and Enrollment were only invoked once, the overall execution time of the IBADConBE scheme is mainly determined by Initialize, Join, Leave, Encrypt and Decrypt algorithms. We note that, under the above settings, the simulation results in this paper are consistent with those in [18]. Hence, in this paper, we only need to briefly describe the simulation results. As shown in Figure 2, when the group size ranges from 3 to 180, Initialize costs from 0.25 s to 0.98 s, the running time of Join for the new wireless node ranges from 0.16 s to 0.58 s while the running time of Join for each group member is from 0.35 s to 0.79 s. We note that the execution time of the above algorithms is largely influenced by the group size. The execution time of Encrypt and Decrypt algorithms slightly increases with the group size. Particularly when the group size is 180, the overall running time of Encrypt and Decrypt is still less than 0.2 s. This result demonstrates that the IBADConBE scheme can be stably and efficiently implemented, even in large groups.

The scalability of the original IBADConBE scheme is quite a new and interesting investigation for us. In fact, our scheme is scalable and can support a larger group size. A general idea is to divide a large group into several subgroups so that the scheme could be effectively applied into each subgroup. We note that the execution time of the encrypt algorithm will increase a little accordingly since a sender has to encrypt a message for multiple times (for each subgroup, less than 0.2 s is required), but the efficiency of the Decrypt algorithm will not be affected.

## 8. Conclusions

In this paper, our main focus was to formalize the security analysis of the identity-based authenticated dynamic contributory broadcast encryption (IBADConBE) scheme. The IBADConBE scheme achieves various security and functional properties, including authentication, message confidentiality, known-key security, dynamic secrecy, trusted dealer freeness, receiver non-restriction, certificate freeness, and dynamicity. However, the original scheme lacked a formal proof to demonstrate that it captured these security properties. Therefore, we first reviewed the IBADConBE scheme and then designed a security model to capture its security properties. Under this model, we provided concrete security proofs based on the asymmetric variant of the decision *k*-BDHE assumption. Finally, we presented a comparison and simulations to show the efficiency of the IBADConBE scheme. As for future work, it would be interesting to consider penetration testing for networks supporting our model.

## Figures and Tables

**Table 1:** Comparison of security and functional properties.

Design Goals	DAGKA [23]	DConBE [17]	IBADConBE
Authentication	Yes	No	Yes
Message confidentiality	Yes	Yes	Yes
Known-key security	Yes	Yes	Yes
Dynamic secrecy	Yes	Yes	Yes
Trusted dealer freeness	Yes	Yes	Yes
Receiver non-restriction	No	Yes	Yes
Certificate freeness	Yes	No	Yes
Dynamicity	Yes	Yes	Yes

## Data Availability

No data were used to support this study.
